# The Germ Theory

**Published:** 1897-06

**Authors:** 


THE GERM THEORY.—"The harmful results of this theory are many. It diverts the attention of the public from the real cause to a supposed one—a mere attendant symptom. It sets our people to hiding around here and there to escape bacilli, when their minds should be directed to the living of such lives as God ordained they should live, thus maintaining a standard of vitality against which the onslaughts of bacilli are vain. This theory turns our physicians from health directors into vermin slayers; it turns them from physiology to toxicology. They cease instructions for rearing healthy children and excite the innocent public with vain imaginings concerning the invisible bacilli. To the germ theorists is due the charge of 'going off after strange gods.' They inflame the minds of an innocent public with the invisible—the imaginary—when the great need is a knowledge of tangible realities."—*Currie, in American Medical Journal.*

(Copied from Homeopathic Envoy.)

